# The role of lipid metabolism in aging, lifespan regulation, and age‐related disease

**DOI:** 10.1111/acel.13048

**Published:** 2019-09-27

**Authors:** Adiv A. Johnson, Alexandra Stolzing

**Affiliations:** ^1^ Nikon Instruments Melville NY USA; ^2^ BIOAGE Labs Richmond CA USA; ^3^ Loughborough University Loughborough UK

**Keywords:** biomarker, ceramides, fatty acids, healthspan, longevity, phospholipids

## Abstract

An emerging body of data suggests that lipid metabolism has an important role to play in the aging process. Indeed, a plethora of dietary, pharmacological, genetic, and surgical lipid‐related interventions extend lifespan in nematodes, fruit flies, mice, and rats. For example, the impairment of genes involved in ceramide and sphingolipid synthesis extends lifespan in both worms and flies. The overexpression of fatty acid amide hydrolase or lysosomal lipase prolongs life in *Caenorhabditis elegans*, while the overexpression of diacylglycerol lipase enhances longevity in both *C. elegans* and *Drosophila melanogaster*. The surgical removal of adipose tissue extends lifespan in rats, and increased expression of apolipoprotein D enhances survival in both flies and mice. Mouse lifespan can be additionally extended by the genetic deletion of diacylglycerol acyltransferase 1, treatment with the steroid 17‐α‐estradiol, or a ketogenic diet. Moreover, deletion of the phospholipase A2 receptor improves various healthspan parameters in a progeria mouse model. Genome‐wide association studies have found several lipid‐related variants to be associated with human aging. For example, the epsilon 2 and epsilon 4 alleles of apolipoprotein E are associated with extreme longevity and late‐onset neurodegenerative disease, respectively. In humans, blood triglyceride levels tend to increase, while blood lysophosphatidylcholine levels tend to decrease with age. Specific sphingolipid and phospholipid blood profiles have also been shown to change with age and are associated with exceptional human longevity. These data suggest that lipid‐related interventions may improve human healthspan and that blood lipids likely represent a rich source of human aging biomarkers.

## 1 INTRODUCTION

Aging is a complex, multifarious process characterized by changes such as stem cell exhaustion, mitochondrial dysfunction, impaired immune function, reduced autophagy, epigenetic alterations, accumulation of somatic and mitochondrial DNA mutations, aberrant intercellular communication, loss of telomeres, altered nutrient sensing, and impaired protein homeostasis (Lopez‐Otin, Blasco, Partridge, Serrano, & Kroemer, 2013; Singh, Demmitt, Nath, & Brunet, 2019). A large portion of our current, limited understanding of what causes aging comes from lifespan studies in short‐lived model organisms. By identifying genetic, pharmacological, and dietary interventions that both extend and reduce lifespan, we have gleaned that specific molecular mechanisms—like the target of rapamycin (TOR), insulin/insulin‐like growth factor (IGF), and adenosine monophosphate‐activated protein kinase (AMPK) signaling pathways—play integral roles in regulating aging (Singh et al., 2019). The identification of aging biomarkers that change over time has concomitantly helped us to understand what mechanisms underlie aging. For example, nicotinamide adenine dinucleotide (NAD^+^) concentrations decrease during aging and high‐fat diets as well as increase in response to caloric restriction, exercise, and fasting (Verdin, 2015). Moreover, NAD^+^ supplementation extends lifespan in mice (Zhang et al., 2016) as well as in yeast and worms (Verdin, 2015). This biomarker data (i.e., that NAD^+^ levels decrease with age) preceded the lifespan data and paved the way for studies exploring the effects of NAD^+^ repletion on aging.

Due to the sheer amount of time and cost required to validate a study in humans, the bulk of our aging and lifespan data come from shorter‐lived yeast, worms, flies, and rodents. With the exception of research showing that caloric restriction improves health and survival in rhesus monkeys (Mattison et al., 2017), little aging work has been done in longer‐lived organisms. The bulk of our understanding regarding aging comes from genetic experiments in model organisms (Singh et al., 2019), and we do not yet know how similar or dissimilar human aging is. For example, genome‐wide association studies searching for longevity‐related variants have found a lack of association with many genes known to extend lifespan in simpler animals (de Magalhaes, 2014). This is likely due to major biological differences between these organisms and humans as well as the limited genetic diversity of laboratory animal strains. As such, it is probable that a large portion of aging interventions proven in the laboratory will not yield significant clinical effects in humans (de Magalhaes, 2014). Therapies that are evolutionarily conserved between different model organisms are, however, more likely to have a therapeutic effect in *Homo sapiens*. Caloric restriction, for example, extends lifespan or improves health in every organism tested—including radically disparate animals such as mosquitoes (Joy, Arik, Corby‐Harris, Johnson, & Riehle, 2010) and humans (Kraus et al., 2019; Most, Tosti, Redman, & Fontana, 2017).

Rather than screen every lifespan‐extending intervention in humans to better understand how human aging works, another approach would be to utilize aging biomarkers. Biomarkers that strongly correlate with aging, lifespan, and healthspan can teach us about which processes are involved in human aging. They can also help us understand, independent of an individual's chronological age, how old a patient is biologically. Clinically, this could be used as an important health assessor. For example, Fleischer et al recently generated and analyzed a large dataset of genome‐wide RNA‐seq profiles of human dermal fibroblasts (Fleischer et al., 2018). These fibroblasts were derived from 133 people aged one to 94 years old. By developing an ensemble machine learning method, they were able to estimate an individual's age to a median error of four years. Testing in ten progeria patients revealed that this transcriptomic approach was capable of predicting accelerated aging (Fleischer et al., 2018). These data are impactful as they suggest that, with sufficient biomarker knowledge, patient senescence could be accurately measured by looking at objective, computer‐analyzed parameters. In the clinic, this would enable precision medicine by giving doctors the ability to make patient‐specific decisions based on their aging state. Put differently, a patient's true biological age could be accurately ascertained instead of making assumptions based on their chronological age. Currently, generalized recommendations are provided given average outcomes associated with different age groups. Robust biomarkers would also allow us to rapidly test the efficacy of rejuvenative interventions in humans (Mahmoudi, Xu, & Brunet, 2019).

Myriad types of aging biomarkers exist. They can take the form of physiological and clinical data such as white blood cell count, absolute monocyte count, blood pressure, body mass index, resting heart rate, forced expiratory volume, gait speed, and grip strength (Burkle et al., 2015; Sebastiani et al., 2017; Xia, Chen, McDermott, & Han, 2017). As an example of how useful one of these biomarker parameters can be, grip strength is highly predictive of mortality, morbidity, and future disability (Leong et al., 2015). Biomarkers can also manifest as analyzed genomic, epigenetic, transcriptomic, and proteomic data. The epigenetic marker DNAm PhenoAge, which is comprised of DNA methylation information from 513 different CpGs, was shown to strongly correlate with age in every tissue tested and to be predictive of all‐cause mortality as well as the age‐related diseases cancer and Alzheimer's disease (Levine et al., 2018). By performing transcriptomic analyses, this marker was also associated with an increased activation of pro‐inflammatory pathways as well as a decreased activation of DNA damage response genes (Levine et al., 2018). Efforts are currently underway to initiate a clinical trial that will utilize DNA methylation information to assess the efficacy of various antiaging interventions (Mitteldorf, 2019). Biomarkers can additionally manifest as molecules such as carbohydrates, apolipoproteins, glycoproteins, hormones, cytokines, and lipids (Burkle et al., 2015; Sebastiani et al., 2017; Xia et al., 2017). Interleukin‐6, for instance, is a pro‐inflammatory cytokine and glycoprotein that increases in concentration with age (Maggio, Guralnik, Longo, & Ferrucci, 2006). This age‐related increase in interleukin‐6 fits into our current understanding that the immune system gets progressively dysregulated with age and that unhealthy inflammation contributes to senescence. The upregulation of the interferon response pathway, for example, occurs during aging in multiple tissues from mice as well as in other vertebrate species such as rats, African turquoise killifish, and humans (Benayoun et al., 2019).

Ideally, a robust and practical biomarker would be one that incurs a low monetary cost and can be measured safely, repeatedly, and easily. Blood draws are especially appealing because they are inexpensive, simple, low risk, and can be taken as needed throughout a patient's lifetime. While several biomarker studies have focused on protein‐based markers, the advancement of metabolomic techniques has made it feasible to look closely into a large array of metabolites. Metabolomic lipids and lipid‐related proteins represent a large, rich source of potential biomarkers that are easily measured in the blood. Compounds in lipid metabolism can take many forms, such as phospholipids, triglycerides, waxes, steroids, and fatty acids. They also play diverse physiological roles, such as forming cell membranes and lipid rafts (Pike, 2003) as well as exerting powerful cell signaling effects (Sunshine & Iruela‐Arispe, 2017). Lipids are perhaps the most well‐known for the paramount roles they play in both the storage and mobilization of energy.

Although lipids have been traditionally treated as detrimental and as simply associated with age‐related diseases, numerous studies have shown that lipid metabolism potently regulates aging and lifespan. Jové et al, for example, assessed the plasma lipidomic profiles of 11 different mammalian species with longevities varying from 3.5 to 120 years (Jove et al., 2013). They found that a lipidomic profile could accurately predict an animal's lifespan and that, in particular, plasma long‐chain free fatty acids, peroxidizability index, and lipid peroxidation‐derived product content are inversely correlated with longevity (Jove et al., 2013). Similarly, Jobson et al scanned the genomes of 25 different species and reported that genes involved in lipid composition had undergone increased selective pressure in longer‐lived animals (Jobson, Nabholz, & Galtier, 2010). Evidence from animals with extreme longevity also links lipid metabolism to aging. The ocean quahog clam *Arctica islandica*, an exceptionally long‐lived animal that can survive for more than 500 years, exhibits a unique resistance to lipid peroxidation in mitochondrial membranes (Munro & Blier, 2012). The bowhead whale, another complex animal with extreme longevity that can live longer than 200 years, has lens membranes that are especially enriched with phospholipids. This unique enrichment is thought to at least partially underlie its uncanny resistance to the age‐related lens disease of cataracts (Borchman, Stimmelmayr, & George, 2017). Naked mole rats, which enjoy remarkably long lifespans and healthspans for rodents, have a unique membrane phospholipid composition that has been theorized to contribute to their exceptional longevity (Mitchell, Buffenstein, & Hulbert, 2007). The importance of lipids in lifespan is further confirmed by the ability of lipid‐related interventions to enhance longevity in model organisms (Huang, Withers, & Dickson, 2014).

The goal of this review was to assess the potential of lipids or lipid‐related proteins to function as biomarkers of aging and to affect aging. To do this, we highlight how alterations in lipid metabolism can impact lifespan and age‐related disease. We discuss how these lipid‐related interventions are distinct from those made by altering canonical aging pathways and also highlight lipid‐associated signatures that correlate with extreme human longevity. Based on the existing data, we believe that lipids are a promising source of human aging biomarkers and that, clinically, they may be able to effectively determine a patient's biological age. We also believe that lipid‐related interventions represent a promising clinical strategy for improving human healthspan and ameliorating age‐related disease. Lastly, we propose aspects of lipid metabolism that could be clinically targeted to elongate the period of healthy life in humans. The ability of lipid‐specific interventions to elongate both lifespan and healthspan in animal models demonstrates that, rather than being simply associated with age‐related disease, lipid metabolism is a direct and potent regulator of aging.

## 2 LIFESPAN EXTENSION VIA LIPID‐RELATED INTERVENTIONS

**Figure 1:**
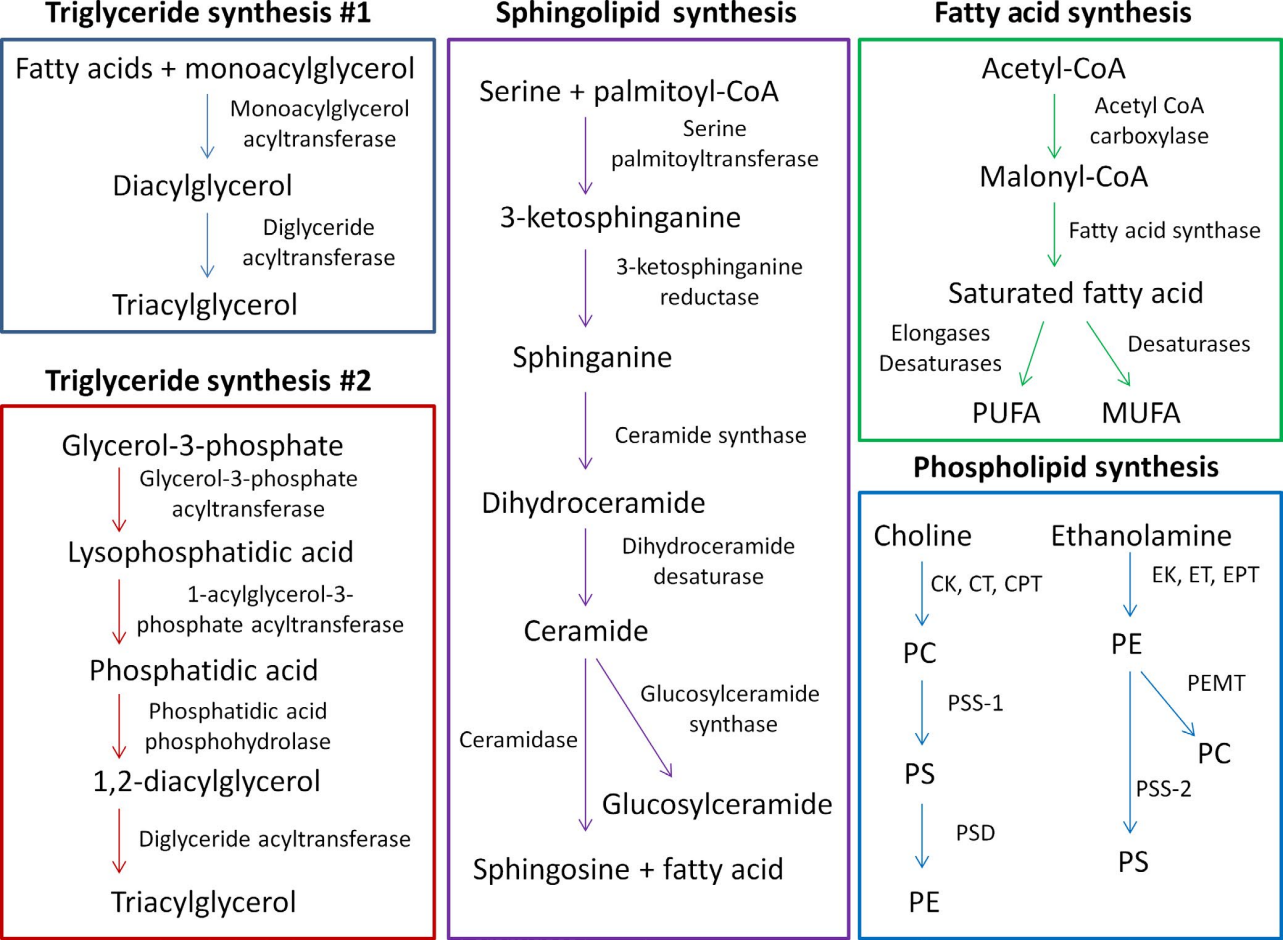
Various aging‐relevant lipid synthesis pathways. The biosynthesis pathways for triglycerides (two different pathways), sphingolipids, fatty acids, and phospholipids are visually summarized. CK, choline kinase; CPT, CDP‐choline:1,2‐diacylglycerol cholinephosphotransferase; CT, CTP‐phosphocholine cytidylyltransferase; EK, ethanolamine kinase; EPT, CDP‐ethanolamine:1,2‐diacylglycerol ethanolaminephosphotransferase; ET, CTP‐phosphoethanolamine cytidylyltransferase; MUFA, monounsaturated fatty acid; PC, phosphatidylcholine; PE, phosphatidylethanolamine; PEMT, phosphatidylethanolamine methyltransferase; PS, phosphatidylserine; PSD, phosphatidylserine decarboxylase; PSS‐1, phosphatidylserine synthase‐1; PSS‐2, phosphatidylserine synthase‐2; PUFA, polyunsaturated fatty acid

One of the most effective ways to understand aging is to assess what interventions can modify lifespan. Different dietary, genetic, pharmacological, and surgical lipid‐related interventions have been shown capable of extending lifespan in model organisms such as worms (Tables 1 and 2), flies (Table 3), and rodents (Table 4). Although several lipid‐related therapies can boost longevity in yeast (Huang et al., 2014), we focus on aging studies in multicellular animals in this review. In this section, we limit our discussion to specific interventions that prolong longevity. In the subsequent section, we delve into associated mechanisms as well as observed trends between these life extension studies. Mentioned lipid synthesis pathways that are relevant to aging are visually summarized in Figure 1. The specifically highlighted pathways are triglyceride synthesis (Ahmadian, Duncan, Jaworski, Sarkadi‐Nagy, & Sul, 2007; Shi & Cheng, 2009), sphingolipid synthesis (Gault, Obeid, & Hannun, 2010), fatty acid synthesis (Jump, 2011; Wakil, Stoops, & Joshi, 1983), and phospholipid synthesis (Vance, 2015; Figure 1). We have additionally created a table that briefly summarizes the primary function of each genetically targeted lipid‐related protein implicated in lifespan regulation (Table 5).

**Table 1:** Lipid‐related nongenetic interventions that extend lifespan in *Caenorhabditis elegans*

Intervention	% Lifespan increase	Relevant observations	Reference
Administration of α‐lipoic acid	24.2	Attenuated hydrogen peroxide levels Enhanced chemotaxis in older worms	Brown et al. (2006)
Administration of α‐lipoic acid	21	Conferred thermal stress resistance	Benedetti et al. (2008)
Feeding with the royal jelly fatty acid 10‐hydroxy‐2‐decenoic acid	12	Life extension was independent of the insulin signaling transcription factor DAF‐16	Honda et al. (2011)
Dietary supplementation with ω‐6 polyunsaturated fatty acids (arachidonic acid or di‐homo‐γ‐linoleic acid)	15.7 for arachidonic acid 17.3 for di‐homo‐γ‐linoleic acid	Inactivation of autophagy reverses the life extension effect Fasting induces the expression of the lysosomal lipase *lipl‐4*, which leads to an enrichment of ω‐6 polyunsaturated fatty acids	O'Rourke et al. (2013)
Administration of fish oil containing eicosapentaenoic acid and docosahexaenoic acid	9.6	Large amounts of fish oil shortened lifespan Fish oil increased lipid peroxide levels in a dose‐dependent manner	Sugawara et al. (2013)
Administration of ketone body ß‐hydroxybutyrate	~20	Increased thermotolerance Decreased α‐synuclein aggregation and delayed amyloid‐β toxicity Life was not extended in a genetic model of dietary restriction	Edwards et al. (2014)
Supplementation with oleoylethanolamide	15.4	Constitutive expression of the lysosomal lipase LIPL‐4 increased the abundance of oleoylethanolamide	Folick et al. (2015)
Supplementation with the lignan matairesinol	25	Tested lignans upregulated the expression of DAF‐16 and JNK‐1	Su and Wink (2015)
Dietary supplementation with monounsaturated fatty acids (oleic, palmitoleic, or cis‐vaccenic acid)	20.98 for oleic acid 18.98 for palmitoleic acid 8.67 for cis‐vaccenic acid	Monounsaturated fatty acid accumulation is required for lifespan extension in H3K4me3 methyltransferase‐deficient worms	Han et al. (2017)
Dietary supplementation with α‐linolenic acid	~30	Life extension required the transcription factors NHR‐49/PPARα and SKN‐1/Nrf2 Additional treatment with the oxylipin 9(S)‐HpOTrE further increases lifespan	Qi et al. (2017)
Supplementation with the lignan sesamin	9.5	Life extension occurs via the SIRT1, TOR, and AMPK signaling pathways	Nakatani et al. (2018)
Treatment with phosphatidylcholine	28.8	Extended life under conditions of oxidative stress Reduced fertility Delayed decline in age‐related motility Accumulation of DAF‐16 in the nucleus Protection against amyloid‐β toxicity	Kim et al. (2019)

**Table 2:** Lipid‐related genetic interventions that extend lifespan in *Caenorhabditis elegans*

Intervention	% Lifespan increase	Relevant observations	Reference
RNAi against the yolk lipoprotein VIT/vitellogenin (*vit‐2* or *vit‐5*)	21–24.4 for *vit‐2* 9.7–21.5 for *vit‐5*	Downregulated in *daf‐2* (−) worms and upregulated in *daf‐16* (−) worms	Murphy et al. (2003)
RNAi knockdown of the ceramide synthase gene *hyl*‐1	14–31	Neither deletion nor overexpression of *hyl*‐1 resulted in life extension	Tedesco et al. (2008)
Constitutive expression of the lysosomal lipase LIPL‐4	24	Long‐lived worms are lean Lipid hydrolysis is induced via decreased insulin signaling	Wang et al. (2008)
RNAi knockdown of elongation of fatty acid protein 1 (*elo‐1*), elongation of fatty acid protein 2 (*elo‐2*), or the fatty acid desaturase *fat‐4*	11 for *elo‐1* 8 for *elo‐2* 15 for *elo‐1* and *elo‐2* 25 for *fat‐4*	Knockdown of both elongases yielded a greater lifespan effect than either elongase alone Depletion of *fat‐4* produced the most significant life extension Gene knockdown was accompanied by increased resistance to oxidative stress	Shmookler Reis et al. (2011)
Overexpression of fatty acid amide hydrolase *faah‐1*	9.1–60	Reduced levels of *N*‐acylethanolamines Resistance to thermal stress Life extension required the Foxa transcription factor PHA‐4 *N*‐acylethanolamine supplementation suppressed life extension	Lucanic et al. (2011)
Inactivation of acid sphingomyelinase‐3 (*asm‐3*)	19 (RNAi knockdown) 14 (mutant worms)	Promotes dauer arrest Life extension depend on the functions of *daf‐18/PTEN* and *daf‐16/FOXO* Inactivation of *age‐1/PI 3‐kinase* further extends lifespan	Kim and Sun (2012)
Functional loss of the ceramide synthase genes *hyl‐1* and *lagr‐1*	21.4	Knockdown of the autophagy‐associated gene *ATG‐12* abolished the longevity effect Increased number of autophagosomes Reduced feeding and reproduction Increased heat resistance	Mosbech et al. (2013)
Small interfering RNAs and pharmacological inhibitors directed against glucosylceramide synthase, serine palmitoyltransferase, dihydroceramide desaturase, or neutral/acidic ceramidase	40 for glucosylceramide synthase 33 for serine palmitoyltransferase 40 for dihydroceramide desaturase 40 for neutral/acidic ceramidase	Slowed development rate Worms fed a yolk diet rich in sphingolipids exhibited a reduced lifespan Silencing of neutral sphingomyelinase shortened lifespan and accelerated development	Cutler et al. (2014)
Overexpression of diacylglycerol lipase	12–13	Diacylglycerol lipase mutants exhibit a shortened lifespan Strains with less lipase activity show reduced resistance to oxidative stress	Lin et al. (2014)
RNAi against the yolk lipoprotein VIT/vitellogenin (*vit‐1/2, vit‐3, vit‐4*, and *vit‐5*)	16–40	Induced autophagy and lysosomal lipolysis Lifespan is reduced by VIT overexpression Required the nuclear hormone receptors NHR‐49 and NHR‐80	Seah et al. (2016)
Overexpression of *fat‐7* in the intestine	14.96–17.55	Increased fat accumulation Supplementation with oleic acid did not further extend lifespan in these transgenic worms	Han et al. (2017)

**Table 3:** Lipid‐related interventions that extend fruit fly or mosquito lifespan

Species	Intervention	% Lifespan increase	Relevant observations	Reference
*Drosophila bipectinata*	Feeding with various concentrations of the lipophilic butylated hydroxytoluene	19.04 for males 26.08 for females	Decreased rate of lipid peroxidation	Sharma and Wadhwa (1983)
*Drosophila melanogaster*	Treatment with α‐lipoic acid	12 for females 4 for males	DJ651‐driven tetanus toxin (DTT) flies treated with α‐lipoic acid exhibited increased survival times	Bauer et al. (2004)
*D. melanogaster*	Overexpression of GLaz, the fly homolog of apolipoprotein D	29	Enhanced resistance to hypoxia Superior walking and climbing posthyperoxia Increased starvation resistance	Walker et al. (2006)
*D. melanogaster*	Overexpression of human apolipoprotein D	40–41	Enhanced protection against hyperoxia, dietary paraquat, and heat stress Reduced age‐associated lipid peroxide accumulation	Muffat et al. (2008)
*D. melanogaster*	Inactivation of *Drosophila* alkaline ceramidase (Dacer) via insertional mutagenesis	54.4 for females 48.3 for males	Increased anti‐oxidative stress capacity Lengthened preadult development time Elevated levels of ceramides	Yang et al. (2010)
*D. melanogaster*	Overexpression of the fatty‐acid‐β‐oxidation‐related genes *fatty acid‐binding protein* or *dodecenoyl‐CoA delta‐isomerase*	81.3 for fatty acid‐binding protein 31.3 for dodecenoyl‐CoA delta‐isomerase	Enhanced tolerance to oxidative stress and starvation Activation of dFOXO signal	Lee et al. (2012)
*D. melanogaster*	Adult fat body overexpression of the histone deacetylase *Sir2*	~13	Transcriptional profiles suggest a role for *Sir2* in regulating lipid droplet biology	Hoffmann et al. (2013)
*D. melanogaster*	RNAi double knockdown of LDL receptor‐related protein 1 and LDL receptor‐related protein 2	Not reported (lifespan curves are shown only for females)	Larval growth is slowed and pupariation is delayed AKT is less phosphorylated Decrease in the number of lipoprotein‐positive neurons	Brankatschk et al. (2014)
*D. melanogaster*	Overexpression of diacylglycerol lipase or knockdown of diacylglycerol kinase	72	Diacylglycerol lipase mutants exhibit a shortened lifespan and a reduced tolerance to oxidative stress	Lin et al. (2014)
*Anopheles stephensi* and *Aedes aegypti*	Transgenic overexpression of a myristoylated and active form of Akt in the fat body	14–47 for *A. aegypti* 15–45 for *A. stephensi*	Activation of the downstream signaling molecules forkhead box O and p70 S6 kinase Increased expression of the fat body vitellogenin	Arik et al. (2015)
*D. melanogaster*	Restricting dietary yeast during development	Up to 145	Suppression of toxic lipids underlies life extension Toxic lipids can shorten male and female lifespan	Stefana et al. (2017)

**Table 4:** Lipid‐related interventions that extend rodent lifespan

Species	Intervention	% Lifespan increase	Relevant observations	Reference
*Mus musculus*	Adipose‐specific insulin receptor knockout mice	18	Reduced fat mass and protection against age‐related obesity	Bluher et al. (2003)
*M. musculus*	Overexpression of human apolipoprotein D	41.6 or 27.5, depending on the dose of paraquat	Increases survival under oxidative stress Prevents the rise of brain lipid peroxides postoxidant treatment	Ganfornina et al. (2008)
*Rattus norvegicus domesticus*	Surgical removal of visceral fat	Not reported (estimated to be ~20% of the longevity effect induced by caloric restriction)	Reduced incidence of severe renal disease Similar rates of tumor incidence	Muzumdar et al. (2008)
*M. musculus*	Mice with additional copies of *Pten*, an inhibitor of the insulin signaling pathway	16 in males 9 in females	Lower cancer incidence Increased energy expenditure, reduced adiposity, and hyperactive brown adipose tissue Protection from insulin resistance and steatosis	Ortega‐Molina et al. (2012)
*M. musculus*	Deficiency of the triglyceride synthesis enzyme acyl‐CoA:diacylglycerol acyltransferase 1	25	Female mice enjoyed protection from age‐related increases in tissue triglycerides, white adipose tissue inflammation, and body fat Middle‐aged mice displayed reduced fecundity and decreased levels of circulating insulin growth factor 1	Streeper et al. (2012)
*M. musculus*	Knockout of the ubiquitin‐like gene *FAT10*	20	Higher metabolic rate, markedly reduced adiposity, and the preferential use of fat as fuel Decreased triglyceride content Enhanced insulin sensitivity	Canaan et al. (2014)
*M. musculus*	Treatment with the steroid 17‐α‐estradiol (4.8 mg/kg)	12 in males	Median, but not maximum, lifespan was increased. Female lifespan was unaffected	Harrison et al. (2014)
*M. musculus*	Treatment with the steroid 17‐α‐estradiol (14.4 mg/kg)	19 in males	Both median and maximal lifespan were increased. Female lifespan was unaffected	Strong et al. (2016)
*M. musculus*	Feeding mice an isocaloric ketogenic diet (89% kcal from fat)	13.6 in males	Improved motor function and memory in aged mice Preservation of muscle mass Reduced tumor incidence Tissue‐dependent regulation of mTORC1 signaling	Roberts et al. (2017)
*M. musculus*	Knockdown of the phospholipase A2 receptor *Pla2r1* in a mouse model of progeria	No statistically significant difference in survival compared with controls, although maximum lifespan was increased in mice lacking *Pla2r1*	Improved grip strength Increased bone mineral content Reduced number of rib fractures Decreased trabecular separation	Griveau et al. (2018)
*M. musculus*	Adipose tissue‐specific overexpression of nicotinamide phosphoribosyltransferase	13.4 in females	Aged transgenic mice display improvements in wheel running activity, sleep quality, glucose tolerance, glucose‐stimulated insulin secretion, and photoreceptor function	Yoshida et al. (2019)

**Table 5:** Functions of different lipid proteins that regulate organismal aging

Lipid protein	Function
Acid sphingomyelinase	Breaks down sphingomyelin into ceramide and phosphorylcholine
Apolipoprotein D	Lipoprotein that transports lipids throughout the body
Ceramidase	Hydrolyzes ceramide into sphingosine
Ceramide synthase	Utilizes sphingoid base and acyl‐CoA substrates to catalyze the formation of ceramides
Diglyceride acyltransferase	Utilizes the substrates diacylglycerol and acyl‐CoA to form triglycerides
Diacylglycerol lipase	Hydrolyzes diacylglycerol into 2‐arachidonoylglycerol
Dihydroceramide desaturase	Converts dihydroceramide into ceramide via the insertion of a 4,5‐trans‐double bond into the sphingolipid backbone of dihydroceramide
Dodecenoyl‐CoA delta‐isomerase	Catalyzes the degradation of long‐chain fatty acids during beta‐oxidation
Fatty acid amide hydrolase	Degrades endogenous signaling lipids in the fatty acid amide family, including *N*‐acylethanolamines
Fatty acid‐binding proteins	Intracellular lipid chaperones
Fatty acid desaturase	Creates a carbon–carbon double bond in a fatty acid by removing two hydrogen atoms
Fatty acid elongase	Extends the carbon chain length of a fatty acid
Glucosylceramide synthase	Transfers glucose to a ceramide
Low‐density lipoprotein‐receptor‐related protein	Cell‐surface endocytic receptor that binds extracellular ligands and targets them for intracellular degradation
Lysosomal lipase	Hydrolase that breaks down fats (e.g., cholesterol and triglycerides) within the lysosome
Phospholipase A2 receptor	Transmembrane protein that can bind to secreted phospholipase A2
Serine palmitoyltransferase	Condenses palmitoyl CoA and serine to form 3‐ketodihydrosphingosine
VIT/vitellogenin	Yolk lipoprotein that delivers cholesterol to oocytes

### 2.1 Nematodes

Different lipid‐related nongenetic (Table 1) and genetic (Table 2) interventions are capable of extending lifespan in *Caenorhabditis elegans*, including the supplementation with fatty acids. In response to fasting, O'Rourke, Kuballa, Xavier, and Ruvkun (2013) found that expression of lysosomal lipase *lipl‐4* was induced, which in turn led to an enrichment of ω‐6 polyunsaturated fatty acids (PUFAs). Direct supplementation of the ω‐6 PUFAs arachidonic acid and di‐homo‐γ‐linoleic acid in culture media promoted starvation resistance and extended animal lifespan. Inactivation of autophagy reversed this increase in lifespan, suggesting that autophagy underlies ω‐6 PUFA‐induced life extension (O'Rourke et al., 2013). Similarly, PUFA treatment with α‐linolenic acid can increase lifespan in a dose‐dependent manner. Oxidized α‐linolenic acid generates oxylipins, and the oxylipin 9S‐hydroperoxy‐10E,12Z,15Z‐octadecatrienoic acid further increases longevity in α‐linolenic acid‐treated worms (Qi et al., 2017). Feeding with 10‐hydroxy‐2‐decenoic acid, a fatty acid component of honeybee royal jelly, similarly increases lifespan (Honda et al., 2011). This fatty acid additionally confers oxidative and thermal stress tolerance (Honda et al., 2015). Dietary supplementation with the monounsaturated fatty acids (MUFAs) oleic, palmitoleic, or cis‐vaccenic acid is also sufficient to increase lifespan. In addition, the accumulation of MUFAs underlies the life extension observed in worms with a deficiency in H3K4me3 methyltransferase, an important epigenetic enzyme (Han et al., 2017). The administration of fish oil containing the PUFAs eicosapentaenoic acid and docosahexaenoic acid has also been reported to enhance longevity, though too much fish oil had the effect of shortening lifespan (Sugawara, Honma, Ito, Kijima, & Tsuduki, 2013).

Other studies have reported nematode life extension in response to treatments with specific, nonfatty acid substances (Table 1). The plant lignan matairesinol, which acts as an adiponectin receptor agonist, was documented to extend mean lifespan by 25% in *C. elegans* (Su & Wink, 2015). Sesamin, which can inhibit delta 5 desaturase (Shimizu et al., 1991), is yet another lignan that has been reported to boost longevity (Nakatani et al., 2018). The compound α‐lipoic acid has been reported to regulate lipid metabolism via the deacetylase sirtuin 1 (Chen, Kang, Wang, & Lee, 2012). α‐lipoic acid, which is derived from the fatty acid octanoic acid (Solmonson & DeBerardinis, 2018), increases thermal stress resistance and elongates life in *C. elegans* (Benedetti et al., 2008). It additionally attenuates H_2_O_2_ levels and improves chemotaxis (Brown, Evans, & Luo, 2006). The histone deacetylase inhibitor and ketone body β‐hydroxybutyrate extend mean lifespan by 20% and increase thermotolerance in *C. elegans*. Relevant to the age‐related neurodegenerative diseases Alzheimer's disease and Parkinson's disease, treatment with this ketone body additionally decreases α‐synuclein aggregation and delays amyloid‐β toxicity (Edwards et al., 2014). Nematodes treated with phosphatidylcholine showed an analogous protection against amyloid‐β toxicity. They additionally displayed longer lifespans under conditions of oxidative stress (Kim, Kim, Park, & Park, 2019).

Nematode lifespan can also be elongated via RNAi knockdown (Table 2). The yolk lipoprotein VIT/vitellogenin is capable of transporting cholesterol to oocytes. Gene silencing of various VIT genes (*vit‐1/2*, *vit‐3*, *vit‐4*, and* vit‐5*) boosts longevity, induces lysosomal lipolysis and autophagy, and increases neutral lipid accumulation (Seah et al., 2016). These data corroborate a prior report from the Cynthia Kenyon Laboratory, which found that RNAi knockdown against either *vit‐2* or *vit‐5* enhances longevity (Murphy et al., 2003). Using RNAi to inhibit elongation of fatty acid protein 1 (*elo‐1*), elongation of fatty acid protein 2 (*elo‐2*), or the fatty acid desaturase *fat‐4* can also extend nematode lifespan as well as increase resistance to oxidative stress. Elongase enzymes are responsible for extending carbon chains, while desaturase enzymes work to create a carbon–carbon double bond by removing two hydrogen atoms from a fatty acid (Shmookler Reis et al., 2011). RNA interference against the ceramide synthase gene *hyl‐1* is another route to life extension (Tedesco, Jiang, Wang, Jazwinski, & Johnson, 2008). Separate, relevant work has shown that *hyl‐1* mutants are more resistant to anoxia than normal animals (Menuz et al., 2009). Using small interfering RNAs against the sphingolipid synthesis enzymes glucosylceramide synthase, serine palmitoyltransferase, dihydroceramide desaturase, and neutral/acidic ceramidase extends lifespan and slows development. Genetically modified serine palmitoyltransferase 1 (*sptl‐1*) mutant worms also lived longer. Conversely, a sphingolipid‐rich egg yolk diet accelerated development and shortened lifespan. Fatty acid chain desaturation and elongation in many sphingolipid species also increased during development and aging (Cutler, Thompson, Camandola, Mack, & Mattson, 2014). Further cementing a role for ceramides and worm aging, RNAi knockdown against acid sphingomyelinase‐3 (ASM‐3), an enzyme that produces ceramide by hydrolyzing sphingomyelin, extends animal lifespan. Genetically inactivating *asm‐3* by introducing a mutation also extended life, though it did so to a reduced degree compared with RNAi‐treated worms (Kim & Sun, 2012).

Several studies have found that genetically modifying lipid genes enhance longevity (Table 2). In response to dietary restriction, there is a reduction in the number of *N*‐acylethanolamines, which are lipid‐derived signaling molecules. Inducing an *N*‐acylethanolamine deficiency via transgenic overexpression of fatty acid amide hydrolase, an enzyme that degrades *N*‐acylethanolamines and other fatty acid amides, is sufficient to extend *C. elegans* lifespan and increase thermal stress resistance. Transgenic worms with extra copies of this hydrolase had reduced levels of palmitoleoyl ethanolamide, linoleoyl ethanolamide, eicosapentaenoyl ethanolamide, and arachidonoyl ethanolamide (Lucanic et al., 2011). Nematode longevity can also be increased via constitutive expression of the lysosomal lipase LIPL‐4, which is an enzyme that hydrolyzes fats such as cholesterol and triglycerides. These transgenic worms were lean and had fewer lipid droplets as well as decreased fat storage (Wang, O'Rourke, & Ruvkun, 2008). Subsequent work found that the constitutive expression of the lysosomal lipase LIPL‐4 increased the abundance of lipid oleoylethanolamide. This lipid promotes longevity and binds directly to the proteins LBP‐8 and NHR‐80 (Folick et al., 2015). Lipolysis mediated by LIPL‐4 was also found to work interdependently with autophagy to prolong lifespan in *C. elegans* (Lapierre, Gelino, Melendez, & Hansen, 2011; Lapierre, Melendez, & Hansen, 2012). Overexpression of the distinct lipid hydrolase diacylglycerol lipase is another route to life extension and additionally promotes resistance to oxidative stress (Lin et al., 2014). Functional loss of the ceramide synthase genes *hyl‐1* and *lagr‐1* via genetic deletion also extends nematode lifespan. This aging effect is abrogated if the autophagy‐associated gene *ATG‐12* is knocked down, and in worms lacking *hyl‐1* and *lagr‐1*, an increased number of autophagosomes are observed. These nematodes additionally display increased heat resistance as well as reduced feeding and reproduction (Mosbech et al., 2013). This work corroborates the previously mentioned data, which reported that RNA interference against *hyl‐1* prolongs life (Tedesco et al., 2008). Intestinal overexpression of the fatty acid desaturase enzyme FAT‐7 is another path to life extension in *C. elegans*. In addition to living longer, these transgenic worms accumulated more fat compared with controls (Han et al., 2017).

### 2.2 Fruit flies

Lipids also impact lifespan in fruit flies (Table 3) and life extension can be induced by several different types of interventions, including dietary changes. Restricting dietary yeast during development in *Drosophila melanogaster* can more than double median lifespan, and the degree of life extension depends on the adult diet posteclosion. Larvae that were fed a low‐yeast diet and that were subsequently fed a low‐yeast diet, a high‐yeast diet, or a low‐yeast, high‐glucose diet as adults showed median lifespan increases of 20%–30%, 70%–90%, and up to 145%, respectively. An important mechanism underlying this life extension is the suppression of toxic lipids dubbed autotoxins. These toxic lipids are shed into the environment and can shorten both male and female *Drosophila* lifespan (Stefana et al., 2017). In contrast to the negative effects of autotoxins, dietary supplementation with various concentrations of the lipophilic compound and antioxidant butylated hydroxytoluene can increase both median and maximum lifespans in *Drosophila bipectinata*. Using the thiobarbituric acid test, it was also shown that flies fed butylated hydroxytoluene exhibited a decreased rate of lipid peroxidation compared with controls (Sharma & Wadhwa, 1983). The compound α‐lipoic acid, which was previously reported to prolong life in worms (Benedetti et al., 2008; Brown et al., 2006), also extends life in *D. melanogaster* and does so in both females and males (Bauer, Goupil, Garber, & Helfand, 2004).

Separate experiments have shown that gene inactivation via RNAi knockdown or insertional mutagenesis can boost longevity in *Drosophila* (Table 3). Glial‐specific double RNAi knockdown against the low‐density lipoprotein (LDL), receptor‐related protein 1 (LRP‐1), and the LDL receptor‐related protein 2 (LRP‐2) extends lifespan. In addition, larval growth is slowed and pupariation is delayed. These receptors were expressed in glial cells and responsible for moving lipoprotein across the blood–brain barrier. Knockdown against these receptors approximately halved the numbers of neurons positive for lipoprotein (Brankatschk, Dunst, Nemetschke, & Eaton, 2014). Both LRP‐1 and LRP‐2 are part of the low‐density lipoprotein receptor family, which is comprised of endocytic, cell‐membrane receptors that facilitate the lysosomal degradation of various extracellular ligands (Spuch, Ortolano, & Navarro, 2012). Inactivation of *Drosophila* alkaline ceramidase, or Dacer, via insertional mutagenesis, is sufficient to extend lifespan and boost anti‐oxidative stress capacity. This life extension comes with a reproductive trade‐off where preadult development time is lengthened. Inactivation of *Dacer* also increased the level of most ceramide species containing either C_14_SPH or C_16_SPH (Yang et al., 2010).

Gene overexpression is another route to life extension in fruit flies (Table 3). Overexpression of diacylglycerol lipase or knockdown of diacylglycerol kinase elongates lifespan and enhances resistance to oxidative stress. Conversely, diacylglycerol lipase mutants experience shorter lifespans and a reduced oxidative stress tolerance. As already mentioned in the prior section, increased activity of this lipase also elongates life in nematodes. Diacylglycerol lipase is known for its role in hydrolyzing diacylglycerol into 2‐arachidonoylglycerol and a free fatty acid. Thus, overexpression of *DAGL* would be predicted to shunt more diacylglycerol into 2‐arachidonoylglycerol (Lin et al., 2014). *Drosophila* lifespan is additionally extended by the overexpression of fatty‐acid‐β‐oxidation‐related gene *fatty acid‐binding protein* or *dodecenoyl‐CoA delta‐isomerase*. This life extension is accompanied by an enhanced tolerance to oxidative stress and starvation. Fatty acid‐binding proteins function as intracellular lipid chaperones, while dodecenoyl‐CoA delta‐isomerase works to catalyze the degradation of long‐chain fatty acids during beta‐oxidation (Lee, Lee, Paik, & Min, 2012). Overexpression of human apolipoprotein D (ApoD), a lipid‐binding protein that promotes resistance to oxidative stress, is sufficient to enhance longevity and reduce age‐associated lipid peroxide accumulation in *Drosophila*. These transgenic flies also enjoyed heightened protection against hyperoxia, dietary paraquat, and heat stress (Muffat, Walker, & Benzer, 2008). Transgenic overexpression of the fly homolog of ApoD, *GLaz*, analogously extends lifespan. Flies overexpressing *GLaz* exhibit an increased resistance to hyperoxia as well as superior walking and climbing abilities following sublethal exposure to hyperoxia. Overexpression of *GLaz* did not significantly alter weight, protein content, or lipid content but it did make flies more resistant to starvation (Walker, Muffat, Rundel, & Benzer, 2006). The more indirect, transgenic overexpression of the histone deacetylase *Sir2* in the adult fat body is another route to enhanced longevity and transcriptional profiling suggests that this gene affects lipid droplet biology (Hoffmann, Romey, Fink, Yong, & Roeder, 2013).

### 2.3 Mosquitoes

Although the intervention is less direct, one study has found that a lipid‐related genetic intervention can prolong life in two different species of mosquitoes (Table 3). The laboratory of Michael Riehle has shown that the transgenic overexpression of the kinase Akt1 exclusively in the fat body can increase survivorship in both *Aedes aegypti* and *Anopheles stephensi* mosquitoes (Arik et al., 2015). Akt1 is a member of the canonical insulin/IGF signaling pathway, and its overexpression in *A. aegypti* or *A. stephensi* mosquito species was sufficient to increase survivorship by 14%–47% or 15%–45%, respectively. Elevated expression of this protein kinase also activated the downstream signaling molecules forkhead box O and p70 S6 kinase. Survivorship differences compared to controls were only observed when mosquitoes were fed blood and were abrogated when mosquitoes were fed only sugar. Interestingly, transgenic mosquitoes also showed increased expression of the fat body vitellogenin, which is a precursor protein to egg yolk (Arik et al., 2015). The lack of a trade‐off between reproduction and longevity as well as the enhancement of a reproductive protein tied to prolonged life is noteworthy.

Although the invertebrate fat body combines many of the functions of adipose tissue and liver in vertebrates (Law & Wells, 1989), it is important to note that there are important differences between this organ and adipose fat in more complex animals. While vertebrate fat is known to promote unhealthy inflammation and produce pro‐inflammatory cytokines, such deleterious effects have not yet been reported in the insect fat body (Azeez, Meintjes, & Chamunorwa, 2014). Thus, lipid‐related longevity findings unique to the insect fat body may not reliably translate to vertebrate species and should be interpreted carefully.

### 2.4 Rodents

While not as numerous as the lifespan data in simple organisms (Tables 1, 2, 3), different interventions linked to lipid metabolism have been shown to augment longevity in rodents (Table 4). In rats, the laboratory of Nir Barzilai has shown that surgical removal of visceral fat at 5 months of age significantly increases both mean and maximum lifespan. It also reduces the incidence of severe renal disease (Muzumdar et al., 2008). Prior work from the same laboratory had shown that the removal of visceral fat improves insulin action and delays the onset of diabetes. Interestingly, the extraction of visceral fat did not alter levels of plasma free fatty acids. It did, however, decrease the expression of leptin and tumor necrosis factor‐alpha in subcutaneous adipose tissue (Gabriely et al., 2002). A third, earlier study from the same research group found that increased insulin sensitivity in response to visceral fat removal was accompanied by a marked decrease in the plasma levels of insulin‐like growth factor‐binding protein‐1 (Barzilai et al., 1999).

We previously mentioned that, in *Drosophila*, overexpression of either human ApoD or *GLaz*, the fly homolog of human ApoD, is sufficient to extend lifespan (Muffat et al., 2008; Walker et al., 2006). Suggestive of an evolutionarily conserved anti‐stress mechanism, overexpression of human ApoD is also capable of increasing survival under conditions of oxidative stress in mice (Ganfornina et al., 2008). This overexpression also prevented the rise of brain lipid peroxides postoxidant treatment. In contrast, loss of function of this gene increased the level of brain lipid peroxidation, reduced protection against oxidative stress, and impaired both learning and locomotor abilities (Ganfornina et al., 2008). Separate work by Thomas and Yao showed that, compared to wild‐type mice, *ApoD* KO mice showed a significant increase in saturated fatty acids (16:0 and 18:0), a monoene (16:1), dienes (linoleic acid and eicosadienoic acid), and a hexane (docosatetraenoic acid) (Thomas & Yao, 2007). Further suggestive of an important role for this lipoprotein, elevating ApoD production via adenovirus‐mediated gene transfer can reduce plasma triglyceride levels, increase plasma lipoprotein lipase activity, and enhance the catabolism of triglyceride‐rich particles in mice. In d*b*/d*b* obese mice, plasma levels of ApoD are reduced (Perdomo et al., 2010).

Another path to life extension in mice involves the steroid 17‐α‐estradiol. Dietary supplementation with 17‐α‐estradiol (4.8 ppm) did not significantly impact lifespan in females but resulted in a 12% median lifespan increase in males (Harrison et al., 2014). The National Institute on Aging Interventions Testing Program has confirmed the lifespan effect of 17‐α‐estradiol at a higher dose (14.4 ppm), reporting that it reliably extends median lifespan in males, but not females. The median lifespan increase, pooled from data across different sites, was 19% (Strong et al., 2016). Recent follow‐up work found that, in late life, 17‐α‐estradiol‐treated mice better maintain their body weight, and in males, this weight is associated with larger muscle fibers and heavier skeletal muscles compared with untreated controls (Garratt et al., 2019). Males also exhibit improved rotarod capacity and grip strength at 25 months. These data suggest that this steroidal estrogen compound can attenuate age‐related sarcopenia. Castrated males do not respond to 17‐α‐estradiol, indicating that gonadal hormones at least partially underlie the observed antiaging mechanisms. This same study reported that late‐life function could be improved even if treatment with 17‐α‐estradiol did not start until 16 months (Garratt et al., 2019). Treatment with this steroid additionally alters the metabolic profile of plasma and liver in males, including raising the abundance of different amino acids in the liver. The second estrogenic steroid estriol‐3‐sulfate was also elevated in males. Once more, these changes are either reduced or inhibited by castration (Garratt et al., 2018).

Directly prolonging longevity by genetically modifying a lipid gene has been demonstrated in mice. This was accomplished by creating a deficiency in the triglyceride synthesis enzyme acyl‐CoA:diacylglycerol acyltransferase 1 (DGAT1). Female mice deficient in DGAT1 enjoyed increased mean and maximal lifespans as well as protection from age‐related increases in tissue triglycerides, body fat, and inflammation in white adipose tissue. Fecundity was decreased, and there were reductions in the levels of circulating insulin growth factor 1 and total cholesterol (Streeper et al., 2012). Prior work in these *Dgat1^−/−^* mice had shown that, although these transgenic animals can still synthesize triglycerides, they are lean and exhibit a resistance to diet‐induced obesity. Compared to controls, they displayed an increase in both energy expenditure and activity. Lactation was, however, defective in females (Smith et al., 2000). Despite being leaner, *Dgat1^−/−^* mice consumed more food at baseline and they had higher surface temperatures. When placed in a cold environment, their hyperphagia became more pronounced (Chen, Ladha, Smith, & Farese, 2003). Although a DGAT1 deficiency recapitulates many aspects of caloric restriction—such as reduced adiposity, decreased tissue inflammation, and a lower fecundity—there are key differences. Not only do *Dgat1^−/−^* mice eat more than control mice, but there are key gene expression differences. Streeper et al performed a whole‐genome microarray and pathway analyses on liver samples from calorie‐restricted mice and *Dgat1^−/−^* mice fed ad libitum. They found that, although caloric restriction upregulates DGAT1 expression approximately twofold, approximately seven times as many genes were changed with caloric restriction versus mice with a DGAT1 deficiency. About 100 genes were commonly upregulated or downregulated, however, and these included genes involved in immune responses, inflammation, and the cholesterol biosynthesis pathway (Streeper et al., 2012). Of note, livers from both Snell dwarf (*Pit1^dw/dwJ^*) and ribosomal protein S6 kinase 1 knockout mice (*S6K1^−/−^*) have similarly been reported to exhibit a decrease in cholesterol biosynthesis genes (Boylston et al., 2004; Selman et al., 2009).

Another genetic intervention worth mentioning is the knockdown of the phospholipase A2 receptor (PLA2R1) in a mouse model of progeria (Griveau et al., 2018). *Pla2r1* encodes for a transmembrane protein receptor that binds to secreted phospholipase A2 proteins and regulates various cell signaling processes (Bernard & Vindrieux, 2014; Sukocheva et al., 2019). In progeria mice with premature aging, knockdown of *Pla2r1* significantly decreases several different aging phenotypes. Specifically, *Pla2r1*‐deficient mice exhibited an improvement in grip strength, an increase in bone mineral content, and a reduction in the number of rib fractures. Trabecular separation in vertebral bone was also decreased. The inflammatory marker IL‐8 and the senescence marker p21 were both reduced in bone mRNA in mice lacking *Pla2r1* compared with control progeria mice. Although maximal lifespan was increased in animals with a PLA2R1 deficiency, the difference in survival did not reach statistical significance compared with controls (Griveau et al., 2018).

While less direct, four other lipid‐related genetic approaches have been reported to extend mouse lifespan. These interventions either make an adipose‐specific genetic modification and/or significantly impact lipid metabolism. The earliest of these studies enhanced longevity by specifically knocking out the insulin receptor in just adipose tissue. Fat‐specific insulin receptor knockout (FIRKO) mice have a normal food intake and show increases in mean, median, and maximum lifespans in both males and females. Moreover, their fat mass is reduced and they enjoy protection against age‐related obesity (Bluher, Kahn, & Kahn, 2003). Follow‐up research discovered that FIRKO mice have an increased basal metabolic rate and a higher respiratory exchange ratio. They also show persistently elevated expression of mitochondrial genes involved in glycolysis, tricarboxylic acid cycle, beta‐oxidation, and oxidative phosphorylation in white adipose tissue from mice aged six to 36 months. In contrast to the FIRKO mouse, expression of these mitochondrial genes tended to decline with age in controls (Katic et al., 2007). Mice harboring additional genomic copies of the phosphatase *Pten*, another member of the canonical insulin signaling pathway, also live significantly longer. They have a lower cancer incidence and expend more energy than controls. Their brown adipose tissue is hyperactive and also has higher levels of the uncoupling protein Ucp1, which the authors show is a downstream target of the transcription factor Foxo1. These transgenic mice additionally present with reduced adiposity as well as protection from insulin resistance and steatosis (Ortega‐Molina et al., 2012).

Outside of the insulin signaling pathway, two additional indirect genetic alterations that are relevant to lipids have increased mouse lifespan. The uncoupling protein genes *UCP2* and *UCP3* were upregulated, and lifespan was extended in mice lacking the ubiquitin‐like gene *FAT10* (Canaan et al., 2014). These FAT10‐deficient mice showed a preferential use of fat as fuel, a higher metabolic rate, less triglyceride content, a markedly reduced adiposity, and decreased weight gain. Enhanced insulin sensitivity was observed in metabolic tissues, and both circulating glucose and insulin levels were reduced. In addition to elevated uncoupling, they also exhibited increased AMPK activity and β‐oxidation (Canaan et al., 2014). Renal tubular epithelial cells from *FAT10^−/−^* mice display abrogated activation of TNF‐α‐induced NF‐κB and a concomitantly reduced induction of NF‐κB‐regulated genes (Gong et al., 2010). Common types of chronic kidney disease are associated with the upregulation of FAT10 in humans (Gong et al., 2010), and *FAT10* gene expression was reported to be upregulated in the tumors of 90% of patients with hepatocellular carcinoma. The *FAT10* gene is also upregulated in gastrointestinal and gynecological cancers (Lee et al., 2003). These data suggest that the upregulation of *FAT10* promotes disease, while a deficiency in *FAT10* delays aging and improves healthspan. A separate indirect genetic approach increased levels of the pro‐longevity metabolite NAD^+^ by overexpressing nicotinamide phosphoribosyltransferase in adipose tissue. This intervention extended the median lifespan and delayed age‐associated mortality in female mice. Healthspan was also increased, as evinced by observations of improved physical activity, better sleep quality, higher glucose tolerance, superior glucose‐stimulated insulin secretion, and enhanced photoreceptor function (Yoshida et al., 2019).

A nongenetic, pro‐longevity dietary intervention is the ketogenic diet, which is an extreme form of a low‐carbohydrate diet. Recent work by Roberts et al. (2017) has shown that, when mice are fed isocaloric amounts of either a ketogenic diet (89% kcal from fat), a low‐carbohydrate diet (70% kcal from fat), or a control diet (65% kcal from carbohydrate), male mice in the ketogenic group showed a median, but not a maximum, increase in lifespan. The ketogenic diet was found to further decrease tumor incidence at the time of death and to preserve muscle mass, memory, and motor function in aged mice. Mice fed a low‐carbohydrate diet were the heaviest and had significantly more fat mass compared with mice fed either ketogenic or control diets. Blood ketones were elevated in the ketogenic diet group, and the concentration of free fatty acids was highest in the low‐carbohydrate diet group. Under a ketogenic diet regime, levels of phosphorylated and total acetyl‐CoA carboxylase were decreased, while levels of carnitine palmitoyltransferase 2 and medium‐chain acyl‐CoA dehydrogenase were increased. Total and phosphorylated pyruvate dehydrogenase protein levels were also increased in response to both low carbohydrate and ketogenic diets. Expression levels of the insulin/IGF and mammalian TOR (mTOR) pathways were also uniquely affected by a ketogenic diet (Roberts et al., 2017). Separate work by Newman et al have shown that a cyclical, isoprotein ketogenic diet reduces midlife mortality and preserves memory performance with age in male mice (Newman et al., 2017).

## 3 LIPID‐RELATED AGING MECHANISMS

These collated lifespan and healthspan data (Tables 1, 2, 3, 4) make a lucid argument for a significant role of lipid metabolism in aging and lifespan regulation. When analyzing the published data in worms, flies, and rodents, some interesting trends begin to emerge. In this section, we discuss the aging mechanisms revealed to us from these lifespan studies and expand upon them.

### 3.1 Fatty acids

Four different studies have shown that feeding with PUFAs or MFAs can enhance longevity in nematodes (Table 1). The compound α‐lipoic acid, which is derived from the saturated fatty acid octanoic acid, can similarly extend lifespan in *C. elegans* and *D. melanogaster* (Tables 1 and 3), while fish oil containing eicosapentaenoic acid and docosahexaenoic acid can prolong life in *C. elegans* (Table 1). Thus, treatment with specific fatty acids is capable of modulating longevity. It would be invaluable to learn whether similar treatments can prolong life in more complex animal models, including vertebrates. Suggestive of the ability of fatty acids to impact important parameters in mice, research by Nehra et al. (2012) have shown that the lifelong consumption of an ω‐3 fatty acid‐rich diet can prolong murine reproduction function, while an ω‐6 fatty acid‐rich diet is associated with poor reproductive success. Further studies are warranted to understand why different fatty acids exert different effects and to uncover what mechanisms each fatty acid utilizes. Unsaturated fatty acids have been reported to induce noncanonical autophagy (Niso‐Santano et al., 2015), which may be one mechanism by which fatty acids impact aging.

While direct feeding with fatty acids is sufficient to enhance longevity, overexpression of fatty acid proteins (e.g., fatty acid amide hydrolase or the fatty acid desaturase FAT‐7 in worms and fatty acid‐binding protein or dodecenoyl‐CoA delta‐isomerase in flies) can also yield desirable aging effects (Tables 2 and 3). Conversely, the disruption of fatty acid genes can induce physiological harm. For example, mutant worms with severe PUFA deficiencies harbor neurological and growth defects (Watts & Browse, 2002). A role for fatty acids in regulating aging is further highlighted by important work by Shmookler Reis et al. (2011). By analyzing fatty acid profiles across a panel of nematode mutants spanning a tenfold range of longevities, it was discovered that both fatty acid chain length and susceptibility to oxidation were substantially decreased in the longest‐lived mutants (Shmookler Reis et al., 2011). The authors proposed a functional model by which fatty acid chain length was reduced to maintain membrane fluidity given a reduction in lipid peroxidation substrates. Eicosapentaenoic acid, the longest chain PUFA the authors observed in their study, had a profoundly negative lifespan effect when fed to *C. elegans* worms. While the shorter‐chain saturated fatty acid palmitic acid also reduced lifespan, it had a less dramatic effect compared with the longer‐chain eicosapentaenoic acid (Shmookler Reis et al., 2011). These results are corroborated by findings by Jové et al, which reported an inverse correlation between a mammal's lifespan and its concentration of long‐chain free fatty acids. Longer‐lived mammals had less plasma long‐chain free fatty acids, and conversely, shorter‐lived mammals had more plasma long‐chain free fatty acids. Mammals with greater lifespans also showed a lower peroxidizability index and less lipid peroxidation‐derived product content (Jove et al., 2013). In general, a higher MUFA:PUFA ratio is thought to be less susceptible to oxidation and is also associated with longevity (Schroeder & Brunet, 2015).

Several other studies have coupled fatty acids to aging. In nutrient‐poor environments or under conditions of oxidative stress, *C. elegans* mobilize lipids from the soma to the germline to support fecundity at the cost of survival. This trade‐off is coupled to the activation of the cytoprotective transcription factor SKN‐1 (Lynn et al., 2015). Although the effects of resveratrol are not consistently observed between different organisms, the improved health and survival of mice fed resveratrol while on a high‐fat diet is linked to the decreased expression of fatty acid synthase and the phosphorylation of acetyl‐CoA carboxylase (Baur et al., 2006). The dietary fat source utilized during dietary restriction can also significantly impact mouse lifespan. Calorically restricted mice fed a diet with lard lived longer than those on a diet supplemented with either fish or soybean oil. Lard is high in both saturated and monounsaturated fatty acids, while soybean oil and fish oil are high in ω‐6 and ω‐3 PUFAs, respectively (Lopez‐Dominguez et al., 2015). Although fish oil (rich in ω‐3 PUFAs) does not extend lifespan in mice and has not been shown to enhance longevity or decrease mortality in humans (Aung et al., 2018; de Magalhaes, Muller, Rainger, & Steegenga, 2016), ω‐3 PUFAs are associated with protection of the brain during aging (Denis, Potier, Heberden, & Vancassel, 2015; Derbyshire, 2018) as well as cardiovascular risk protection (Innes & Calder, 2018) and improved clinical outcomes for rheumatic diseases (Akbar, Yang, Kurian, & Mohan, 2017). Certain ω‐6 PUFAs have also been linked to better health outcomes in humans. A meta‐analysis of randomized placebo‐controlled clinical trials revealed that the consumption of supplements containing eicosapentaenoic acid and docosahexaenoic acid was associated with lipid‐lowering, hypotensive, anti‐arrhythmic, and anti‐inflammatory action (AbuMweis, Jew, Tayyem, & Agraib, 2018).

Multiple myeloma has also been linked to fatty acid metabolism. It has been proposed that bone marrow adipocytes support the growth and evolution of cancer cells by providing them with free fatty acids (Masarwi, DeSchiffart, Ham, & Reagan, 2019). Relevantly, it was recently shown that the inhibition of fatty acid transport protein 2 blocks tumor progression in mice and that this protein is upregulated in polymorphonuclear myeloid‐derived suppressor cells, which are pathologically activated neutrophils that contribute to the failure of cancer therapies. The pathological activity of these cells involves the uptake of arachidonic acid and the synthesis of prostaglandin E_2_ (Veglia et al., 2019).

### 3.2 Lipases, lipoproteins, and cholesterol

Studies in nematodes and fruit flies have shown that the duration of life can be extended by overexpressing a lipase enzyme (Tables 2 and 3). Moreover, life extension via increased lysosomal lipase activity has been linked to the antiaging, repair‐associated process of autophagy (Lapierre et al., 2012).

Lipases catalyze the hydrolysis of fats and work to process lipids such as triglycerides and cholesterol. The enzymatic activity and mRNA levels of pancreatic lipase are decreased in older mice, and concomitantly, elderly mice exhibit decreased lipid absorption (Yamamoto et al., 2014). Lipoprotein lipase activity has analogously been reported to decrease with age in rat postural skeletal muscle (Bey, Areiqat, Sano, & Hamilton, 2001), and during physical inactivity, activity of this same lipase is suppressed (Bey & Hamilton, 2003). Inactivity also caused a local reduction in the uptake of plasma triglyceride into muscle as well as a decrease in the concentration of high‐density lipoprotein (HDL) cholesterol. Treadmill walking raised lipoprotein lipase activity ~eightfold (Bey & Hamilton, 2003). Lipoprotein lipase hydrolyzes the triacylglycerol component of lipoproteins, which transport fat molecules throughout the body. Aberrant lipoprotein lipase function is associated with obesity, Alzheimer's disease, infection, insulin resistance, dyslipidemia associated with diabetes, chylomicronemia, and atherosclerosis (Mead, Irvine, & Ramji, 2002). Monoacylglycerol lipase is highly expressed in primary tumors and promotes cancer pathogenesis via regulation of a fatty acid network (Nomura et al., 2010). These data suggest that, broadly, altering the activity of specific fat lipases may delay aging and symptoms of age‐related disease. It would be invaluable to know whether or not overexpressing a lipase could extend lifespan in mice or other vertebrate models.

Lipoproteins also have an important role to play in aging. Not only does inhibition of the yolk lipoprotein VIT/vitellogenin prolong life in worms (Table 2), but the overexpression of the lipoprotein ApoD enhances survival and promotes stress resistance in flies and mice (Tables 3 and 4). In dogs, the expression of the apo‐B, E lipoprotein receptors declines linearly with increasing age. These receptors are capable of binding both the apo‐B‐containing low‐density lipoproteins (LDLs) as well as the apo‐E‐containing cholesterol‐induced HDLs (Mahley, Hui, Innerarity, & Weisgraber, 1981). In rats, plasma cholesterol levels increase with age. This increase can be attenuated by treatment with growth hormone, and this attenuation was presumed to occur via effects on lipoprotein metabolism (Parini, Angelin, & Rudling, 1999). Old mice show an impaired lipid mobilization response to fasting that includes milder fasting‐induced changes in apolipoprotein gene expression compared with young mice (Araki, Okazaki, & Goto, 2004). Lipoproteins have also been correlated with various age‐related ailments. For example, human serum concentrations of lipoprotein(a) are significantly associated with an increased risk of Alzheimer's disease (Solfrizzi et al., 2002) and HDL cholesterol tends to be inversely associated with cancer risk (Pirro et al., 2018). HDL cholesterol and triglycerides have been positively and negatively associated with an increased risk of age‐related macular degeneration, respectively (Colijn et al., 2019). HDL cholesterol is also a predictor of major cardiovascular events in patients treated with statins (Barter et al., 2007). Moreover, interventions that promote positive health outcomes are linked to LDL and cholesterol. Mice treated with the drug metformin, for example, enjoy an extended healthspan and lifespan as well as reduced LDL and cholesterol levels (Martin‐Montalvo et al., 2013).

### 3.3 Triglycerides

One of the lipid‐related, lifespan‐increasing interventions in mice targeted triglyceride synthesis by creating a deficiency in the triglyceride synthesis enzyme acyl‐CoA:diacylglycerol acyltransferase 1 (Table 4). Relatedly, long‐lived mice lacking the ubiquitin‐like *FAT10* gene displayed decreased triglyceride content (Canaan et al., 2014) and an adenovirus‐mediated increase in ApoD, a longevity‐relevant lipoprotein (Tables 3 and 4), reduces plasma triglyceride levels in mice (Perdomo et al., 2010). These data indicate that triglycerides are closely tied to the aging process. By compared metabolic parameters in young and aged mice, Houtkooper et al have shown that aging is accompanied by decreased levels of plasma triglycerides and increased levels of free fatty acids (Houtkooper et al., 2011). The peptide hormone insulin, which is a well‐known regulator of aging, expands *Drosophila* fat cell mass by increasing the number of adipocytes and by promoting triglyceride storage (DiAngelo & Birnbaum, 2009). Linking triglycerides and lipases, the loss of adipose triglyceride lipase function is frequently observed in various types of human cancers. The loss of this lipase in a mouse model was found to induce the spontaneous development of pulmonary neoplasia (Al‐Zoughbi et al., 2016). Mice deficient in senescence marker protein‐30, an androgen‐independent factor that dwindles with age, have shorter lifespans as well as higher levels of total hepatic triglyceride, total hepatic phospholipids, and cholesterol (Ishigami et al., 2004). Adipocytes, which store triglycerides, have also been shown to promote metastatic initiation by sensitizing melanoma cells to the cytokine TGF‐β (Golan et al., 2019). These data make a strong case for triglycerides being highly relevant to aging and age‐related disease. More specifically, a common theme appears to be that elevated levels of triglycerides are associated with physiological dysfunction.

### 3.4 Ceramides and sphingolipids

Sphingolipids, including ceramides, have their own role to play in regulating aging (Tables 1, 2, 3). In nematodes, four different studies were able to extend the lifespan of *C. elegans* by inhibiting sphingolipid machinery (Table 2). These inhibited molecular targets include ceramide synthase genes, a sphingomyelinase, serine palmitoyltransferase, glucosylceramide synthase, dihydroceramide desaturase, and neutral/acidic ceramidase (Table 2). In *D. melanogaster*, inactivation of the ceramidase enzyme *Drosophila* alkaline ceramidase is sufficient to extend lifespan (Table 3). It is interesting that the impairment of sphingolipid/ceramide metabolism can prolong life in two different animal models. Data from rats show that sphingolipid catabolic enzyme activity increases during aging (Sacket, Chung, Okajima, & Im, 2009). Lifespan data from yeast further demonstrate that chronological lifespan can be elongated by reducing the rate of sphingolipid synthesis (Huang et al., 2014). Thus, disrupting the production of specific sphingolipids appears to exert pro‐longevity effects.

There are situations, however, where interfering with the sphingolipid pathway can have detrimental health effects. Ceramide transfer protein is responsible for transferring ceramide from the endoplasmic reticulum to the Golgi complex. *D. melanogaster* flies functionally lacking this protein exhibit an increase in membrane fluidity, reduced protection against oxidative damage, decreased thermal tolerance, and shortened lifespans (Rao et al., 2007). *C. elegans* worms that lack sphingosine kinase have decreased lifespans, smaller brood sizes, and reduced body sizes. They additionally show worse locomotor behavior and neuromuscular function in old age (Chan et al., 2017). It has also been suggested that defects in sphingolipid metabolism contribute to the pathogenesis of different brain disorders, including the age‐related neurodegenerative diseases Alzheimer's disease and Parkinson's disease (Di Pardo & Maglione, 2018). More data are required to understand why some sphingolipid‐targeted interventions are pro‐aging and why others are antiaging.

The ability of sphingolipids to influence aging matches up with their essential biological roles. The release of ceramide by acid sphingomyelinase, for example, is a prerequisite for CD95 signaling and apoptosis induction (Grassme et al., 2001). This is clinically significant as CD95 promotes tumor growth, and conversely, the loss of CD95 reduces both the incidence and size of tumors (Chen et al., 2010). More broadly, ceramides increase in concentration with age in mammals and have been linked to various age‐related ailments, including cancer, type 2 diabetes, neurodegeneration, immune dysfunction, and cardiovascular disease. Ceramide accumulation is also correlated with increased insulin resistance and oxidative stress (Huang et al., 2014). Very recent work by Chaurasia et al. (2019) have shown that the deletion of dihydroceramide desaturase 1 improves insulin resistance and hepatic steatosis in mice. Mechanistically, ceramide was revealed to promote the uptake and storage of lipids and to impair glucose utilization (Chaurasia et al., 2019). Thus, clinical therapies that reduce ceramide concentrations may delay or ameliorate symptoms of aging in humans.

### 3.5 Phospholipids

It is intriguing that, in a mouse model of progeria, deleting the phospholipase receptor PLA2R1 improved specific healthspan parameters (Table 4). In the same study, knockdown of this receptor was shown to prevent senescence in human fibroblasts (Griveau et al., 2018). PLA2R1 is associated with both cancer suppression (Bernard & Vindrieux, 2014) and idiopathic membranous nephropathy (Coenen et al., 2013). More broadly, PLA2R1 is thought to be a regulator of various biological processes, including pro‐inflammatory signaling, apoptosis, senescence, and autoimmunity (Sukocheva et al., 2019). Bowhead whales and naked mole rats, two animals characterized by exceptional longevity, both exhibit unique phospholipid profiles (Borchman et al., 2017; Mitchell et al., 2007). The disruption of lipid hydrolases that regulate phospholipid metabolism has also been shown to decrease lifespan in worms (Park et al., 2018), flies (Kinghorn et al., 2015; Kunduri et al., 2014), and mice (Shinzawa et al., 2008). Conversely, treatment with phosphatidylcholine prolongs life in *C. elegans* under conditions of oxidative stress (Kim et al., 2019). While it has yet to be shown that modulating phospholipid machinery can lead to a statistically significant increase in vertebrate lifespan, these data all suggest that phospholipids are highly relevant to aging.

### 3.6 Ketogenic diet

In the prior section, we discussed that treatment with the ketone body ß‐hydroxybutyrate extends lifespan and increases thermotolerance in *C. elegans* (Edwards et al., 2014), while a ketogenic diet prolongs lifespan and healthspan in mice (Newman et al., 2017; Roberts et al., 2017). In worms, ß‐hydroxybutyrate has been proposed to extend life via two different antiaging pathways, the first of which would inhibit histone deacetylases and lead to increased DAF‐16/FOXO activity. The second pathway involves the mitochondrial metabolism of ß‐hydroxybutyrate, which would increase the production of reactive oxygen species via increased citric acid cycle metabolism and electron transport chain activity. This would activate the SKN‐1/Nrf2 antioxidant response pathway and promote longevity (Edwards, Copes, & Bradshaw, 2015). In the original lifespan study, markers of neurodegenerative disease were attenuated in response to treatment with ß‐hydroxybutyrate (Edwards et al., 2014). A more recent paper by Manzo et al. (2018) have shown that, in a *Drosophila* model of amyotrophic lateral sclerosis, a significant decrease and increase were observed in the levels of ß‐hydroxybutyrate and carnitine conjugated long‐chain fatty acids, respectively. Feeding flies either ß‐hydroxybutyrate or medium‐chain fatty acids improved locomotor function (Manzo et al., 2018). These data indicate that this ketone body can exert neuroprotective effects in two different animal models.

The ketogenic diet has been described as a biochemical model of fasting and works by producing ketone bodies (e.g., β‐hydroxybutyrate, acetoacetate, and acetone) from fats when glycogen stores have been depleted in the liver. Ketone bodies are thought to affect neurons by inducing changes in metabolism, epigenetics, and signaling (Fedorovich, Voronina, & Waseem, 2018). Interestingly, two separate studies have shown that a ketogenic diet preserves memory performance during aging in male mice (Newman et al., 2017; Roberts et al., 2017). Given these data and that a ketogenic diet has been used to treat human epilepsy for almost a century (Boison, 2017), it seems reasonable to hypothesize that ketone bodies exert neuroprotective effects. Indeed, a recent case study found that a ketogenic diet rescued cognition in a 71‐year‐old female patient with a dual diagnosis of mild Alzheimer's disease and metabolic syndrome. The patient was heterozygous for the epsilon 4 allele of *ApoE* (Morrill & Gibas, 2019). It would be interesting to see whether, rather than a ketogenic diet, treatment with specific ketone bodies is sufficient to improve healthspan parameters and exert neuroprotective effects in vertebrate animal models.

### 3.7 Canonical aging biology pathways

Although the specific mechanisms by which lipid interventions affect lifespan are largely unknown, it is clear that different interventions exhibit disparate levels of overlap with canonical aging pathways, such as dietary restriction. For example, ß‐hydroxybutyrate does not extend life under dietary restriction in *C. elegans*, suggesting that this ketone body is a dietary restriction mimetic (Edwards et al., 2015). Similarly, the royal jelly component 10‐hydroxy‐2‐decenoic acid does not elongate life in *eat‐2* *C. elegans* mutants, suggesting the mechanism of action for this fatty acid overlaps with dietary restriction (Honda et al., 2015). Treatment with *N*‐acylethanolamine eicosapentaenoyl ethanolamide suppresses dietary restriction‐induced life extension in worms. Moreover, enhanced longevity in response to overexpression of the fatty acid amide hydrolase *faah‐1* requires the dietary restriction‐relevant Foxa transcription factor PHA‐4 (Lucanic et al., 2011). Expression levels of VIT lipoprotein and lysosomal lipases are decreased and increased, respectively, in response to dietary restriction (Seah et al., 2016). Full life extension via dietary restriction also involves the expression of fatty‐acid‐β‐oxidation‐related genes (Lee et al., 2012). Enhanced longevity in response to the loss of the two ceramide synthase genes *hyl‐1* and *lagr‐1* requires the transcription factors PHA‐4/FOXA, DAF‐16/FOXO, and SKN‐1 (Mosbech et al., 2013). The life extension in response to surgical removal of visceral fat in ad libitum‐fed rats was significantly less than the life extension observed in rats that were calorically restricted. This suggests that caloric restriction works, at least in the part, by pathways that are distinct from those that are affected by the removal of visceral fat (Muzumdar et al., 2008). Although some overlap exists, the significantly different gene expression profiles in calorically restricted mice versus *Dgat^−/−^* mice indicate that this is yet another longevity mechanism that is not identical to caloric restriction (Streeper et al., 2012).

A few of the lifespan‐promoting studies discussed in the prior section investigated how a given intervention interacted with the canonical insulin/IGF pathway. The laboratory of Cynthia Kenyon has shown that the absence of *daf‐2* in nematodes leads to the downregulation of the yolk lipoprotein genes *vit‐2* and *vit‐5* (Murphy et al., 2003). Separate research efforts have demonstrated that long‐lived *age‐1* worms exhibit decreased transcript levels of the fatty acid elongase genes *elo‐1*, *elo‐2*, and *elo‐5* as well as the fatty acid desaturase *fat‐4* (Shmookler Reis et al., 2011). The longevity effect reported in response to knockdown of ASM‐3 depended on the functions of the insulin signaling genes *daf‐16/FOXO* and *daf‐18/PTEN*. Double mutant animals with both an *asm‐3* deficiency and an *age‐1/PI 3‐kinase* deficiency were even longer‐lived (Kim & Sun, 2012). Reduced *daf‐2* activity upregulates the lysosomal lipase *lipl‐4,* and RNAi against *lipl‐4* partially suppresses the longevity of *daf‐2* mutants (Wang et al., 2008). Life extension generated by the overexpression of fatty‐acid‐β‐oxidation‐related genes activates dFOXO signal (Lee et al., 2012), and RNAi knockdown of *LRP‐1* and *LRP‐2* decreases how much AKT is phosphorylated (Brankatschk et al., 2014). Similarly, phosphatidylcholine treatment promotes the nuclear accumulation of DAF‐16 (Kim et al., 2019). Conversely, the HYL‐2 ceramide synthase acts independently of DAF‐2 (Menuz et al., 2009) and 10‐hydroxy‐2‐decenoic acid‐induced life extension in *C. elegans* is not dependent on DAF‐16 or DAF‐2 (Honda et al., 2015, 2011).

The TOR and AMPK signaling pathways have also been implicated in lipid lifespan studies. For example, the royal jelly component 10‐hydroxy‐2‐decenoic acid failed to extend lifespan in already long‐lived *daf‐15* mutants. Since *daf‐15* is a target of TOR, the authors conclude that this fatty acid overlaps with the TOR signaling pathway (Honda et al., 2015). DAF‐15/raptor has, however, been reported to be a point of convergence for both the insulin/IGF and TOR signaling pathways (Jia, Chen, & Riddle, 2004). Overexpressing diacylglycerol lipase decreases the phosphorylation levels of S6 kinase, a target of TOR, while creating a transgenic mutant of this protein elevates the phosphorylation levels of S6 kinase (Lin et al., 2014). In a long‐lived TOR pathway mutant with a defect in the worm ortholog of S6 kinase, levels of the lipid eicosapentaenoyl ethanolamide were decreased. Direct treatment of these worms with eicosapentaenoyl ethanolamide suppressed their longevity phenotype, thereby implicating *N*‐acylethanolamines in their extended lifespan (Lucanic et al., 2011). Inhibition of TOR in *C. elegans* was also found to induce the expression of the lysosomal lipase *lipl‐4* (Lapierre et al., 2011). In mice, mTORC1 signaling was regulated in a tissue‐dependent manner by a ketogenic diet (Roberts et al., 2017). Like TOR, AMPK is a metabolic sensor that regulates the synthesis, oxidation, and lipolysis of lipids (Wang, Liu, Zhai, Zhang, & Tian, 2018). In the mouse study which showed that FAT10 knockout mice live longer, the authors noted that these mice had increased AMPK activity in skeletal muscle (Canaan et al., 2014).

Future studies should investigate more closely how different pro‐longevity lipid interventions overlap with each other as well with the classical aging pathways of dietary restriction, insulin/IGF, TOR, and AMPK.

## 4 LIPID BIOMARKERS OF AGING IN HUMANS

### 4.1 Genetic lipid signatures correlated with extreme human longevity

Genome‐wide association studies have identified multiple genetic factors that are correlated with exceptional longevity. Given the ability of lipid‐related interventions to modulate lifespan and healthspan in model organisms (Tables 1, 2, 3, 4), lipid‐related genes would be expected to be associated with longer lifespans in humans.

This is indeed the case. Work by Atzmon et al. (2006) have shown that, among centenarians, homozygosity for the −641C allele in the *APOC3* promoter (rs2542052) is significantly higher compared with controls. This genotype was associated with lower serum levels of APOC3, a favorable pattern of lipoprotein levels and sizes, a lower prevalence of hypertension, and greater insulin sensitivity (Atzmon et al., 2006). A separate study involving 338 centenarians implicated both ApoE and angiotensin‐converting enzyme in human aging. The epsilon 4 allele of ApoE was significantly less common in centenarians compared with controls. Conversely, the epsilon 2 allele of ApoE was significantly more common in centenarians (Schachter et al., 1994). Ryu, Atzmon, Barzilai, Raghavachari, and Suh (2016) have shown that the ε3/ε4 ApoE genotype is markedly depleted in centenarians, while the ε2/ε3 genotype is substantially enriched. The epsilon 4 allele of ApoE is also a risk variant for late‐onset neurodegenerative diseases and is thought to contribute to the pathogenesis of Alzheimer's disease via multiple different pathways (Yamazaki, Painter, Bu, & Kanekiyo, 2016). Given that overexpression of human ApoD or *GLaz*, the fly homolog of ApoD, was shown to confer antiaging benefits in flies and mice (Tables 3 and 4), it is interesting that apolipoproteins are also associated with greater human longevity. This suggests that the protective effects of apolipoproteins are highly evolutionarily conserved and that clinical interventions targeting apolipoproteins could improve human healthspan.

In addition to ApoE, the *PON1* gene encoding paraoxonase/arylesterase 1 is reportedly associated with human longevity and is thought to impact lipid metabolism (Lescai, Marchegiani, & Franceschi, 2009). Tindale et al compared a group of healthy individuals aged 85 or over to random midlife controls and identified that both the *ε4 ApoE* allele and the haptoglobin *HP2* allele were less common among the healthy, aged group. Moreover, a network analysis of candidate longevity genes revealed that lipid and cholesterol metabolism was a common theme. Although it did not reach statistical significance (*p* = .052), the lipoprotein A gene *LPA* showed an interaction with the insulin signaling‐relevant *FOXO3* (Tindale, Leach, Spinelli, & Brooks‐Wilson, 2017). A separate study also identified lipoprotein A as being longevity‐associated (Joshi et al., 2017). Rs7844965, located in an intron of the lipid hydrolase *EPHX2*, was linked with increased human lifespan in a group of UK Biobank participants (Pilling et al., 2017). Genome‐wide association of 1 million parental lifespans has shown that gene pathways involving lipid proteins and homeostasis, synaptic function, and vesicle‐mediated transport are enriched for lifespan variation (Timmers et al., 2019). In addition, a group of Ashkenazi Jews with exceptional longevity (mean age of 98.2 years) and their offspring were reported to have an increased frequency of homozygosity for the codon 405 valine allele of the gene *CETP*, which encodes for cholesteryl ester transfer protein (Barzilai et al., 2003).

### 4.2 Lipidomic analyses of extreme human longevity

A few studies have done broad, lipidomic analysis to assess the relationship between aging and a large array of different lipids. For example, measurements of 128 lipid species using liquid chromatography coupled to mass spectrometry previously identified 19 lipid species associated with familial longevity in women. The authors compared the plasma lipidome of offspring of nonagenarians (a person between 90 and 99 years) to non‐nonagenarian controls. Although no significant differences were observed for men, female offspring exhibited increased levels of either sphingomyelin or phosphocholine species as well as lower levels of long‐chain triglycerides and phosphoethanolamine. Longevity was also associated with a higher ratio of MUFAs over PUFAs (Gonzalez‐Covarrubias et al., 2013). Interestingly, many long‐lived organisms or mutants have a decreased ratio of PUFA to MUFA (Papsdorf & Brunet, 2019). Separate work utilizing NMR metabonomics and shot‐gun lipidomics found that centenarians display unique changes in biosynthesis compared with elderly controls. In particular, phospholipids and sphingolipids were identified as putative markers and modulators of healthy aging (Montoliu et al., 2014). Recent lipidomic work by Jové et al. have reported that a fatty acid profile resistant to lipid peroxidation is associated with extreme longevity. Extreme longevity was associated with a higher saturated fatty acid content as well as a lower content of unsaturated fatty acids, such as PUFAs. Longer lifespans were also correlated with a higher average fatty acid chain length. The authors also proposed that specific lipid species of ceramides are biomarkers of extreme longevity (Jove et al., 2017). Even more recently, Wong et al have used lipidomics to discover that their “oldest old” subjects over 95 years of age exhibited globally low levels of lipids. Women and men also showed sex‐related age differences in their plasma lipid levels. For example, women had higher levels of LDL cholesterol, HDL cholesterol, and total cholesterol. Sphingomyelin and docosahexaenoic acid‐containing phospholipid levels were also higher in females (Wong et al., 2019).

Although more studies and data are required to better understand how the lipidome changes with age, these few studies harbor shared findings. Two different lipidomic screens reported that sphingomyelin levels increase with age for women (Gonzalez‐Covarrubias et al., 2013; Wong et al., 2019). Two of these papers reported that human longevity is associated with lower unsaturated fatty acid content (Gonzalez‐Covarrubias et al., 2013; Jove et al., 2017) and two different studies identified sex‐specific age differences in lipid content (Gonzalez‐Covarrubias et al., 2013; Wong et al., 2019). All three studies linked sphingolipids to aging (Gonzalez‐Covarrubias et al., 2013; Jove et al., 2017; Wong et al., 2019). Broader lipidomic screens are warranted to better understand additional plasma lipids that with age and how plasma lipids uniquely change for men and women.

### 4.3 Triglycerides as blood biomarkers of aging

Other nonlipidomic studies have analyzed whether or not a single lipid or few different lipids are useful as biomarkers of aging or age‐related disease. Multiple papers have identified plasma triglycerides, the lipids stored in fat cells that make up most of body fat, as a potential blood aging biomarker. For example, Parthasarathy et al have reported that triglycerides levels are inversely correlated with cognitive function in nondemented elderly adults (Parthasarathy et al., 2017). Triglycerides have been reported to increase progressively with age and have been actively proposed as a biomarker of aging (Xia et al., 2017). Triglyceride levels increase in older patients and are thought to be a significant risk factor for coronary artery disease, particularly in women (LaRosa, 1997). These data suggest that triglycerides have the potential to be a useful lipid biomarker. However, it is important to note that genetically predicted triglyceride levels have been reported to be unassociated with either frailty and longevity in elderly populations (Liu et al., 2017). Ergo, while most studies indicate that triglycerides increase with age (Papsdorf & Brunet, 2019), it remains to be determined whether or not triglyceride levels can accurately predict parameters of aging.

### 4.4 Lipoproteins and cholesterol as blood biomarkers of aging

Given everything mentioned heretofore, it should be unsurprising that ApoE plasma levels have been proposed as a human aging biomarker and have been reported to strongly associate with cardiovascular mortality (Mooijaart et al., 2006). The related LDL apolipoprotein B and LDL cholesterol reportedly both increase with age, and these increases are linked to a progressively reduced fractional catabolic rate of LDL apolipoprotein B. Stimulation of hepatic LDL receptor expression via the cholesterol‐lowering medication cholestyramine in six old males was sufficient to increase the catabolic rate to levels identified in younger subjects. These data indicate the LDL increase with age occurs due to a reduced capacity for its removal (Ericsson et al., 1991).

Several studies have examined how LDL and HDL change over time, and some of the results are conflictive. A systematic review by Wirth et al. (2017) assessed various markers of human sedentary behavior in the elderly. They identified a positive correlation for LDL and ApoA1 as well as a negative correlation for HDL with older sedentary behavior (Wirth et al., 2017). Offspring of individuals with exceptional longevity have significantly larger LDL and HDL particle sizes as well as a lower prevalence of cardiovascular disease, hypertension, and metabolic syndrome (Barzilai et al., 2003). Ashkenazi Jewish offspring of centenarians showed fewer and larger LDL particles compared with their same‐aged partners. No differences in HDL particle phenotypes were reported (Heijmans et al., 2006). Larger LDL particle sizes as well as lower triglyceride levels were reported in offspring of nonagenarian siblings compared with controls, which were partners of the offspring. LDL particle size was associated with male longevity, while triglyceride levels were associated with female longevity (Vaarhorst et al., 2011). HDL from older subjects was reported to have an altered composition that impaired its antioxidant properties and overall function. HDL from elderly patients contained less cholesterol and had more sphingomyelin (Holzer et al., 2013). Relatedly, a progressive decline in plasma HDL concentrations has been associated with cognitive dysfunction in centenarians (Atzmon et al., 2002).

Data also suggest that plasma cholesterol may be a viable human aging blood biomarker, though the data are disparate. Both female and male offspring of centenarians reportedly have higher plasma levels of HDL cholesterol compared with controls. Men also exhibited significantly lower LDL cholesterol levels (Barzilai, Gabriely, Gabriely, Iankowitz, & Sorkin, 2001). Work by Weijenberg, Feskens, and Kromhout (1996) found that total cholesterol decreased with age, but HDL cholesterol did not change significantly with age in Dutch men. Kreisberg and Kasim previously concluded that total cholesterol, HDL cholesterol, and LDL cholesterol change uniquely over time for both men and women (Kreisberg & Kasim, 1987). A biomarker signature comprised of multiple different biomarkers, including total cholesterol, was found to be associated with lower morbidity and mortality as well as better physical function (Sebastiani et al., 2017). More comprehensive, systematic analyses are required to better understand the relationship between cholesterol and lipoproteins with aging as well as how this relationship differs between men and women.

### 4.5 Fatty acids and lipid peroxidation as blood biomarkers of aging

Fatty acids and lipid peroxidation have additionally been implicated as potential blood lipid biomarkers of human aging. Given that a higher dietary intake and higher circulating levels of eicosapentaenoic acid and docosahexaenoic acid have been associated with a reduced risk of dementia, Tan et al. analyzed red blood cell levels of these ω‐3 PUFAs in 1,575 dementia‐free patients. Participants with lower docosahexaenoic acid had lower scores on tests for visual memory, abstract thinking, and executive function. Those with docosahexaenoic acid levels in the lowest quartile also had greater white matter hyperintensity and lower total brain volumes. The authors concluded that this PUFA was therefore a candidate marker of brain aging (Tan et al., 2012). By assessing different fatty acids with age, it was reported that plasma saturated, polyunsaturated, and monounsaturated fatty acids increase with age. Concomitant with this, circulating concentrations of IL‐6 and TNF‐α increased, while IL‐10 and TGF‐β1 decreased over time. Certain saturated fatty acids were reported to be associated with changing levels of TGF‐β1 and TNF‐α (Pararasa et al., 2016).

In normal elderly people, a decrease in antioxidants and an increase in lipid peroxidation were reported compared with younger controls. The lipid peroxidation malondialdehyde was highly elevated in older patients with diabetes and hypertension (Akila, Harishchandra, D'Souza, & D'Souza, 2007). Yavuzer et al. (2016) have shown that both hypertension and aging are associated with higher lipid peroxidation in humans. In particular, they identified lipid hydroperoxide and thiobarbituric acid‐reactive substances as sensitive markers for both hypertension and aging in elderly patients (Yavuzer et al., 2016). Aging is additionally associated with an increase in lipid peroxidation in cardiac muscle obtained from 59 patient donors (age range of 8–86 years) with a mean age of 56 ± 12 years (Miro et al., 2000).

### 4.6 Sphingolipids and phospholipids as blood biomarkers of aging

Very little work has explicitly assessed whether or not sphingolipids, including ceramides, are candidate human aging blood biomarkers. Plasma sphingolipids have been proposed as biomarkers for Alzheimer's disease (Mielke & Haughey, 2012) and have also been linked to the age‐related diseases of diabetes, obesity, nonalcoholic fatty liver disease, insulin resistance, and cardiovascular disease (Iqbal, Walsh, Hammad, & Hussain, 2017). The plasma ceramide C16:0 has been associated with a slower gait, an important aging marker of physical function (Wennberg et al., 2018). These data as well as the lipidomic data previously mentioned (Gonzalez‐Covarrubias et al., 2013; Montoliu et al., 2014; Wong et al., 2019) nicely justify further exploring the relationship between blood sphingolipids and human aging. Indeed, a recent study in nondiabetic patients found that higher levels of plasma insulin and an increased HOMA of insulin resistance score were associated with an elevation in plasma ceramides (Lemaitre et al., 2018).

With regard to phospholipids, low plasma levels of lysophosphatidylcholines were found to be associated with impaired mitochondrial oxidative capacity in adults (Semba et al., 2019). Lower levels of blood phospholipids, including the lysophosphatidylcholine 18:2, were separately shown to be highly predictive of memory impairment in older adults (Mapstone et al., 2014). Low plasma levels of lysophosphatidylcholine 18:2 also predict a greater decline of gait speed in the elderly (Gonzalez‐Freire et al., 2019). Patients with cancer, a classical age‐related disease, analogously show a decrease in the concentration of plasma lysophosphatidylcholine (Taylor, Arends, Hodina, Unger, & Massing, 2007). Given these data and that both phosphatidylcholine and phosphatidylethanolamine have been reported to decline with age in model organisms (Papsdorf & Brunet, 2019), phospholipids show substantial potential as blood aging biomarkers in humans. Interestingly, a study by Trabado et al. (2017) have reported that elderly healthy subjects have higher plasma levels of sphingomyelins and phosphatidylcholines compared with young subjects. Although this reinforces the theory that these lipids are connected to aging, it suggests that specific lipids within these families may uniquely increase or decrease with age. It also suggests that other parameters, like patient health or genetic variability, may influence the relationship between a given sphingolipid or phospholipid with age.

## 5 CONCLUDING REMARKS AND FUTURE DIRECTIONS

**Figure 2:**
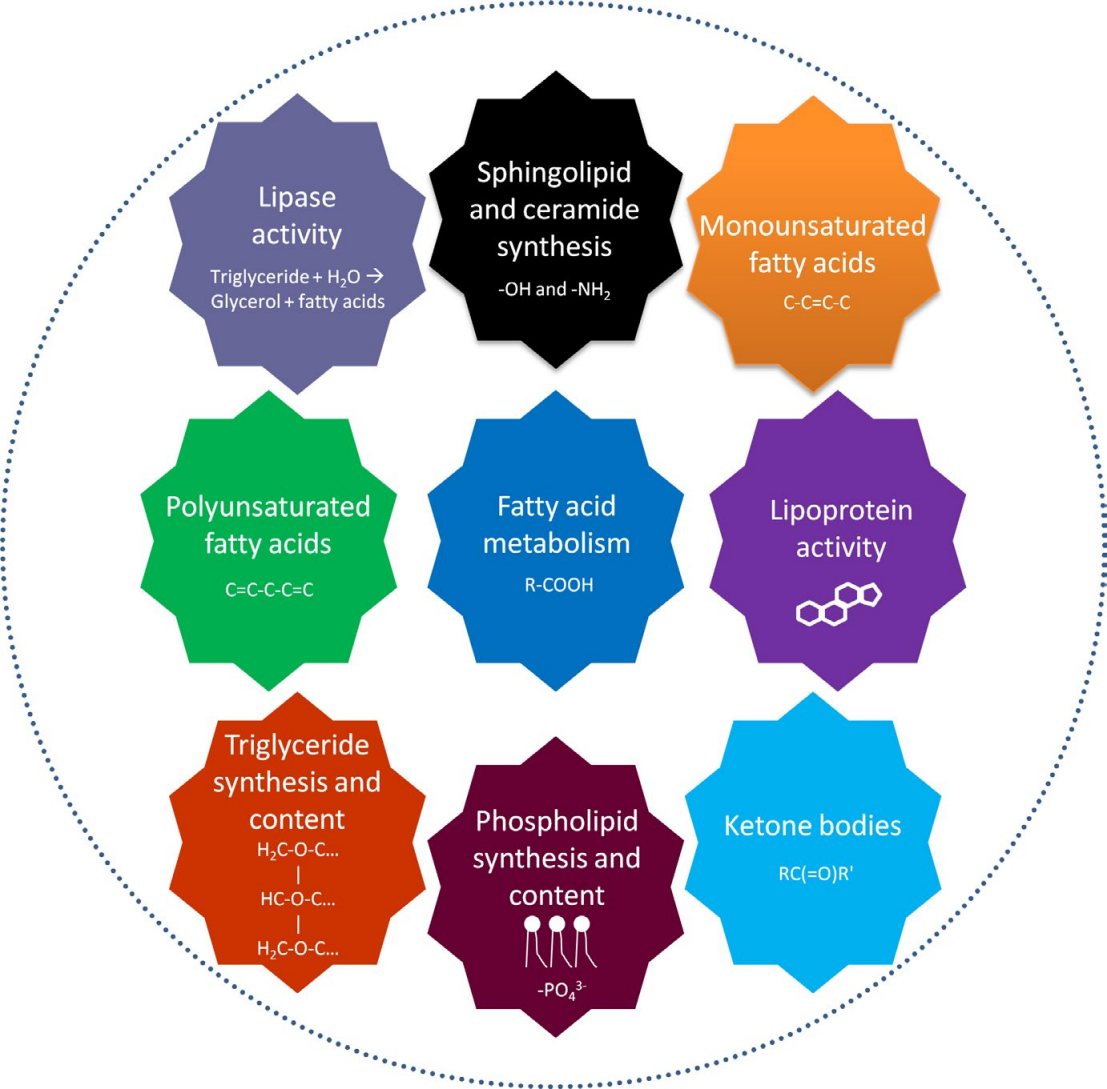
Proposed lipid‐related pathways that could be targeted to extend human healthspan. The overexpression of lysosomal lipase enhances longevity in worms, while the overexpression of diacylglycerol lipase extends lifespan in both worms and flies. Gene inactivation or inhibition of genes encoding the sphingolipid‐relevant sphingomyelinase‐3, glucosylceramide synthase, serine palmitoyltransferase, dihydroceramide desaturase, neutral/acidic ceramidase, or ceramide synthase proteins extends life in *Caenorhabditis elegans*, while the inactivation of alkaline ceramidase increases lifespan in *Drosophila melanogaster*. Longevity can also be increased by feeding specific monounsaturated or polyunsaturated fatty acids to worms, by overexpressing fatty acid amide hydrolase in worms, or by overexpressing fatty acid‐binding protein or dodecenoyl‐CoA delta‐isomerase in flies. The overexpression of apolipoprotein D enhances survival in flies and mice, and the overexpression of the fly homolog of this gene extends lifespan in flies. In worms, RNAi knockdown against the yolk lipoprotein VIT/vitellogenin prolongs life. Survival time can also be elongated by RNAi knockdown against low‐density lipoprotein‐receptor‐related protein 1 and low‐density lipoprotein‐receptor‐related protein 2 in *Drosophila*. Creating a deficiency in the triglyceride synthesis enzyme acyl‐CoA:diacylglycerol acyltransferase 1 boosts longevity in mice and knockdown of the phospholipase A2 receptor improves healthspan parameters in a mouse model of progeria. Relevant to the latter finding, treating worms with phosphatidylcholine boosts longevity. Treating *C. elegans* with the ketone body ß‐hydroxybutyrate or feeding mice with a ketogenic diet additionally extends lifespan. There are likely additional lipid‐related healthspan targets that remain to be elucidated

Although many questions remain to be elucidated, it is clear that lipid metabolism has an imperative role to play in regulating the aging process. Lipid‐related interventions are capable of modulating lifespan in various model organisms. Moreover, specific lipids and lipid‐related molecules have been shown to increase or decrease in an age‐dependent manner. Lastly, lipid‐related genetic markers can strongly correlate with exceptional longevity in humans. These qualities exceed the requirement for a hallmark of aging (Lopez‐Otin et al., 2013) and demonstrate unequivocally that lipid metabolism is intimately connected to the aging process. They additionally highlight several different potential pathways that could be targeted to increase human healthspan (Figure 2). Since many of our proposed target pathways (Figure 2) overlap with each other (e.g., lipase activity and fatty acid metabolism), it would be intriguing to learn what aging mechanisms are shared between each of these targets when they impact longevity.

Future work should aim to better understand the mechanisms that underlie lifespan changes in response to specific lipid‐related interventions in model organisms. Additional research in vertebrate models, such as African turquoise killifish, mice, rats, and Rhesus monkeys, is especially needed. Identifying unique lipid characteristics shared among animals with extreme longevity (e.g., Greenland shark, bowhead whale, giant tortoise, and ocean quahog clam) or theoretical immortality (e.g., planarian flatworms and hydra) would also help illuminate pro‐longevity lipid pathways. Another approach would be to do comprehensive analyses of healthspan parameters and the incidence of age‐related disease in patients being treated with lipid‐relevant pharmacological interventions or patients with lipid‐related genetic mutations. This would help to identify targets and treatments that could be explicitly utilized to elongate human healthspan.

We are also hopeful that lipid signatures could be developed as reliable biomarkers to accurately predict human biological age. Although they are usefully predictive, large human cohort studies represent a current bottleneck and it might be good to think about alternative study designs. If data sharing becomes more common, reanalyzing data may help to glean new insights from existing datasets. Another experimental approach could make use of the many apps that exist to trace daily food intake, composition, and activity. Recruiting people to document daily intake of specific food compounds could be coupled with various measurements to study lipids and aging or aging‐related health outcomes in large human datasets. Given the current data, we are optimistic that certain lipid‐related interventions are capable of extending human healthspan.

## AUTHOR CONTRIBUTIONS

AAJ and AS wrote the manuscript. AAJ compiled the tables and created the figures. Both AAJ and AS contributed to the design of the manuscript.
